# Supplementary material for: Determining the Key Education Priorities Related to Heart Failure Care in Nursing Homes: A Modified Delphi Approach
Source: Healthcare (Basel). 2024 Aug 5;12(15):1546. doi: 10.3390/healthcare12151546 (PMC11311690; doi:10.3390/healthcare12151546)
Supplement: Supplementary file 1 [file healthcare-12-01546-s001.zip › Supplmentary materials S1 - HF Delphi Items (full list).pdf]

Appendix 1: Delphi Survey for first round

These 58 Delphi Items have been generated based on three pieces of clinical guidance and two pieces of empirical research led by the co-authors:

- Standards of Education and Practice for Nurses New to Care Home Nursing published by the Queen's Nursing Institute (QNI) in 2021.
- Heart Failure Association of the European Society of Cardiology heart failure nurse curriculum by the European Society of Cardiology in 2016.
- Heart Failure Specialist Nurse Competency Framework by the Royal College of Nursing (RCN) in 2020.
- A scoping review of the international literature on educational interventions about heart failure in nursing homes by McMahon et al. in 2023.
- A grounded theory on facilitators and barriers to optimising care of residents living with heart failure in nursing homes by Mitchell et al. (Manuscript in preeration)

General Heart Failure Education:

1.1. Nurses working in care homes should receive comprehensive education on heart failure pathophysiology and aetiology.

1.2. Education should cover the different types and stages of heart failure and their clinical manifestations.

1.3. Nurses should be educated on evidence-based guidelines for heart failure management and care.

1.4. Education should emphasise the importance of early recognition and prompt management of heart failure exacerbations.

1.5. Nurses should receive education to have an awareness of assessment tools and diagnostic tests for heart failure evaluation.

Person-Centred Care for Heart Failure Residents:

2.1. Nurses should be trained to provide person-centred care tailored to the individual needs and preferences of heart failure residents.

2.2. Education should focus on promoting communication skills to effectively engage and support residents with heart failure and their families.

2.3. Nurses should learn techniques to assess and address the emotional and psychosocial aspects of heart failure care.

2.4. Nurses should be trained to involve residents with heart failure in policy development to ensure person-centered care.

2.5. Nursing homes should prioritise embedding a policy specifically addressing the needs of heart failure residents.

Medication Management and Adherence:

3.1. Education should cover the management of heart failure medications, including dosage adjustments and monitoring for side effects.

3.2. Nurses should receive training on promoting medication adherence among heart failure residents.

3.3. Education should address the use of diuretics, ACE inhibitors, beta-blockers, MRAs and ARNI (sacubitril/valsartan), and other heart failure medications.

Nutrition and Fluid Management:

4.1. Nurses should be educated on heart-healthy diets and when a fluid restriction is required for heart failure residents.

4.2. Education should emphasise the importance of monitoring fluid balance and recognising signs of fluid overload or dehydration.

4.3. Nurses should receive education and training on dietary modifications to manage sodium in residents with heart failure.

4.4. Nurses should receive education and training on conducting regular assessments for changes to appetite and weight in residents with heart failure, and steps for management.

Symptom Management and Palliative Care:

5.1. Education should cover symptom management strategies for dyspnoea, fatigue, and other heart failure symptoms.

5.2. Nurses should be trained in the provision of a palliative care approach to improve the quality of life for residents with advanced heart failure.

5.3. Education should address end-of-life care discussions and advance care planning for residents with heart failure.

5.4. Nurses should receive education about how to recognise, prevent and manage cachexia in residents with heart failure.

5.5. Nurses should receive education on heart failure management, incorporating high-fidelity simulation as a valuable learning method.

5.6. Nurses should receive education on preventive strategies to reduce heart failure incidence in older care home populations.

Technological Advancements in Heart Failure Management:

6.1. Nurses should be updated on the latest technological advancements for heart failure monitoring and management.

6.2. Education should provide nurses with an understanding of telemedicine and remote monitoring for heart failure residents.

Multidisciplinary Collaboration:

7.1. Nurses should receive training on effective collaboration with other healthcare professionals involved in heart failure care both within primary and secondary care settings.

7.2. Education should emphasise the importance of teamwork and communication within the care home setting.

Cultural Competence in Heart Failure Care:

8.1. Nurses should be educated on providing culturally competent care to residents with heart failure from diverse backgrounds.

8.2. Education should address potential cultural barriers that may impact heart failure management.

Quality Improvement and Evidence-Based Practice:

9.1. Nurses should receive training on quality improvement initiatives to enhance heart failure care in care homes.

9.2. Education should emphasise the use of evidence-based practice to guide shared decision-making in heart failure care.

Self-Care for Heart Failure Residents and Resilience for Nurses:

10.1. Nurses should receive education on promoting exercise as a self-care behaviour to improve outcomes of heart failure residents with heart failure.

10.2. Education should cover the promotion of abstaining from smoking as a self-care behaviour to improve outcomes of heart failure residents with heart failure.

10.3. Nurses should receive education on how to promote self-management and monitoring of symptoms for residents with heart failure.

10.4. Education should cover self-care strategies to prevent burnout and promote resilience among nurses caring for residents with heart failure.

10.5. Nurses should be educated on stress management techniques and coping strategies.

Family and Caregiver Support:

11.1. Nurses should be trained to provide education and support to family members and caregivers of residents with heart failure.

11.2. Education should address family dynamics and the impact of heart failure on caregivers.

Emergency Preparedness and Crisis Management:

12.1. Nurses should receive training on emergency preparedness and crisis management for heart failure exacerbations.

12.2. Education should cover the recognition of severe warning signs and appropriate actions during acute heart failure events.

Ethical and Legal Considerations in Heart Failure Care:

13.1. Nurses should be educated on ethical principles related to heart failure care decision-making.

13.2. Education should address legal issues and documentation requirements in heart failure care.

Communication with Primary Care Providers:

14.1. Education should cover effective communication with primary care providers and specialists involved in heart failure care.

14.2. Nurses should learn how to provide concise and accurate reports to primary care providers.

Falls Prevention and Safety:

16.1 Nurses should receive education about the direct and indirect links between heart failure and falls.

16.2. Education should cover falls assessment and prevention strategies for residents with heart failure, considering their increased risk of falls.

16.3. Nurses should learn techniques to promote a safe environment within the care home.

End-of-Life Care and Bereavement Support:

17.1. Nurses should receive training on end-of-life care principles and providing support to other residents and families during the bereavement process.

17.2 Nurses should receive education about the process of advance care planning in the context of caring for residents with heart failure.

17.3. Education should address grief and loss issues in the care home setting.

Education on Research and Innovation:

18.1. Nurses should be informed about ongoing research, quality improvement and innovative approaches in heart failure care.

18.2. Education should encourage nurses to participate in research projects related to heart failure in care homes.

18.3 Education should encourage nurses from care homes to lead research or quality improvement projects at care home settings.

Interprofessional Communication:

19.1. Education should emphasise effective communication and collaboration with non-nursing care home staff such as health care assistants, catering services, domestic care workers and others.

19.2 Education should emphasise effective communication and collaboration with multidisciplinary professionals such as general practitioners, specialist community nurses, primary care nurses and community pharmacists.

Personal and Professional Development:

20.1. Nurses should be encouraged to engage in continuous personal and professional development related to heart failure care.

20.2. Education should address opportunities for advanced education and training in the care home setting.

20.3 Education should facilitate care home nurses to undertake a ‘heart failure’ champion role or link nurse role within their care home setting.

These statements cover various aspects of heart failure education for nurses working in care homes, focusing on person-centred care, symptom management, medication management, multidisciplinary collaboration, and more. Participants in the Delphi survey can rate the importance of each statement using a Likert Scale.
